# Development of novel microsatellite markers to analyze the genetic structure of dog populations in Taiwan

**DOI:** 10.5713/ab.21.0519

**Published:** 2022-03-02

**Authors:** Fang-Yu Lai, Yu-Chen Lin, Shih-Torng Ding, Chi-Sheng Chang, Wi-Lin Chao, Pei-Hwa Wang

**Affiliations:** 1 Key Laboratory of Animal Genetics, Breeding and Bioresources, Department of Animal Science and Technology, College of Bioresources and Agriculture, National Taiwan University, Taipei 10672, Taiwan; 2 Department of Animal Science, Chinese Culture University, Taipei 11114, Taiwan; 3 Department of Animal Industry, Council of Agriculture, Taipei 100212., Taiwan

**Keywords:** Dog, Novel Microsatellite Marker, Population Genetic Analysis

## Abstract

**Objective:**

Alongside the rise of animal-protection awareness in Taiwan, the public has been paying more attention to dog genetic deficiencies due to inbreeding in the pet market. The goal of this study was to isolate novel microsatellite markers for monitoring the genetic structure of domestic dog populations in Taiwan.

**Methods:**

A total of 113 DNA samples from three dog breeds—beagles (BEs), bichons (BIs), and schnauzers (SCs)—were used in subsequent polymorphic tests applying the 14 novel microsatellite markers that were isolated in this study.

**Results:**

The results showed that the high level of genetic diversity observed in these novel microsatellite markers provided strong discriminatory power. The estimated probability of identity (P_(ID)_) and the probability of identity among sibs (P_(ID)sib_) for the 14 novel microsatellite markers were 1.7×10^−12^ and 1.6×10^−5^, respectively. Furthermore, the power of exclusion for the 14 novel microsatellite markers was 99.98%. The neighbor-joining trees constructed among the three breeds indicated that the 14 sets of novel microsatellite markers were sufficient to correctly cluster the BEs, BIs, and SCs. The principal coordinate analysis plot showed that the dogs could be accurately separated by these 14 loci based on different breeds; moreover, the Beagles from different sources were also distinguished. The first, the second, and the third principal coordinates could be used to explain 44.15%, 26.35%, and 19.97% of the genetic variation.

**Conclusion:**

The results of this study could enable powerful monitoring of the genetic structure of domestic dog populations in Taiwan.

## INTRODUCTION

After strong artificial selection for over 150 years, morphological variation has been created in dog populations and more than 400 canine breeds are currently registered worldwide by the Fédération Cynologique Internationale (FCI), and other federations such as the American and British Kennel Clubs [1]. With over 470 million dogs being kept as companion animals worldwide, they were ranked as the leading type of pet in 2018 [2]. In Taiwan, according to survey statistics from the Council of Agriculture, the number of dogs being bred by the end of 2017 had reached 1.78 million. The overall sales revenue of pet-related industries has increased from 15.5 billion New Taiwan dollars (NT$) in the past 10 years to 26.6 billion NT$ (Statistical Bulletin, Ministry of Finance, Republic of China; http://service.mof.gov.tw/File/Attach/86088/File_21588.pdf). The previous roles of companion animals, such as hunting, security, and assistance, have gradually shifted towards them being regarded as family members. With the improvement of the status of pets, owners are increasingly paying attention to their quality of life. Therefore, the demand for various goods and services aimed at companion animals is also increasing. In the early stage of the Taiwan dog-sales market, the characteristics prioritised included the ability to guard, search, attack, and hunt; more recently, these ideals have been superseded by the need to provide companionship and prestige, which have made a recognized pedigree a major factor for buyers. In Taiwan, some legal purebreed grounds used closed flock breeding to keep their dog broodlines, including those three breeds in this study. Especially the Beagles, which have been used to hunt hares in the British Isles for centuries, and which were brought to the United States in 1880 to breed in large numbers. The modern Beagles have been modified to become a pet dog, and were often used as experimental dogs [3]. It has considerable medical research value, and this dog was also cultivated as laboratory animal for medical research in Taiwan.

So far, many dog breeds have been developed in order to meet appearance standards and maintain the purity of their bloodlines. Breeding companies usually adopt inbreeding methods, which can lead to the occurrence of many genetic diseases. Generally, in the natural state—unlike with experimental animals for which it can be essential to reduce individual differences for study purposes—inbreeding approaches have seldom been used, in order to avoid inbreeding depression [4]. The Royal Society for the Prevention of Cruelty to Animals (RSPCA) pointed out that dogs are now subject to more than 300 genetic diseases. Not only do the animals have to bear great suffering, but their owners also experience mental pressures and financial losses [5]. Therefore, in order to maintain high heterozygosity and stability of the genetic background of the entire population, it is necessary to have a reliable breeding system and genetic monitoring [6]. However, in Taiwan, the genealogical and registration data requirements for many breeds of dog are incomplete or remain to be established, and most of the certificates of pedigree produced by breeding sites in the market lack the backing of publicly trusted authorities, so trading disputes arise from time to time. Although trading in companion animals is discouraged in many countries today, the market in Taiwan is booming. In order to prevent companion animals from being afflicted by genetic diseases, in addition to promoting care by and education of owners, an important factor is reducing inbreeding. The Kennel Club has established a breeding certification system for 68 breeds since 2008, with the total number now reaching 222 breeds. Breeders can inquire about the diseases to be screened for each dog and the procedures for obtaining certification. After the puppies are certified by the Kennel Club, the breeders are issued with a puppy sale wallet; this measure not only protects the profits of the owner, but also reduces disputes over the sale of companion animals [7].

Regarding the research on dog microsatellite markers, a lot of information has been discovered in conjunction with the elucidation of DNA sequences [8]. As early as the 1990s, there have been many studies on dog microsatellite markers [9,10]. The application of canine microsatellite markers in modern times has mainly focused on two fields: one is evaluating the genetic structure polymorphism of populations, and the other is proving a platform for individual identification or paternity [11,12]. Wictum et al [13] searched for published dog genome sequences, and selected suitable microsatellite markers based on the stability and high polymorphism that were required for forensic applications. A total of 15 microsatellite markers and a marker related to gender comprise the multiplex system, DogFiler [13]. This is the first dog-identification data system created based on the recommendations of the Scientific Working Group for DNA Analysis Methods (SWGDAM) in the United States. At present, DogFiler has been integrated into forensic casework, and is widely accepted by courts in the United States. Owing to the relatively long period of strong artificial selection, the differentiation between dog breeds has been large. The sequences on both sides of microsatellite markers may have different degrees of variation, making it impossible to perform polymerase chain reaction (PCR) amplification in similar breeds. Even if the amplification is successful, the number of alleles and polymorphisms may be far lower than in the original breeds [14]. Therefore, when conducting research on the population genetics of specific breeds of dog, it is necessary to develop new microsatellite markers.

To date, only a few domestic biotechnology companies in Taiwan use foreign dog microsatellite commercial kits, such as the StockMarks for dogs genotyping kit, for individual genetic analysis or genetic structure analysis at dog breeding sites. So, are they applicable to Taiwan? To our knowledge, there are no relevant published reports on existing dog breeds for reference and analysis. Therefore, the development of microsatellite markers suitable for the companion animal population in Taiwan is a crucial task to establish a molecular-detection platform for domestic dogs. Moreover, such a platform will make it possible to evaluate the inbreeding level of the dog population in Taiwan. In addition to assisting with the formulation of domestic dog breeding-management policies, this could also enhance Taiwan’s positive governance perspectives on animal protection and animal welfare.

## MATERIALS AND METHODS

### Experimental animals and sample collection

In total, five populations of dogs from legal breeding ground were investigated in this study. Blood samples were collected from 113 individuals belonging to these three populations which were popular in Taiwan: 17 bichons (BIs); 14 schnauzers (SCs); 74 Taiwanese beagles (BETs); and eight Japanese beagles (BEJs), two of which were distinguished as group A (BEJAs) and six of which were distinguished as group B (BEJBs) (Table 1). For each dog, 5 mL of blood was drawn from the jugular or cephalic vein. Whole genomic DNA (gDNA) was then extracted with the Genomic DNA Isolation Reagent (GenePure Technology Co., Ltd, Taichung, Taiwan) using the standard phenol-chloroform method. NanoDrop 2000c (Thermo Fisher Scientific Inc., Waltham, MA, USA) detected the DNA concentration and the optical density (OD) 260/280 values between 1.8 and 2.0, and samples were stored in a refrigerator at −20°C for later use. All animal experiments were approved by the Institutional Animal Care and Use Committee of our university (Protocol number: NTU Animal Experiments No. 2012-089).

### Isolation of microsatellite loci

The gDNA was isolated from blood samples taken from one male and one female BI using the following steps, which were slightly modified from the procedure described by Glenn and Schable [15]. The isolated gDNA was partially digested with *Rsa*I and *Xmn*I (NEB, Ipswich, MA, USA) until most of the DNA fragments were between 300 and 1,000 base pairs (bp) in length. Re-naturing of single-strand SuperSNX24 forward (5′GTTTAAGGCCTAGCTAGCAGAATC3′) and SuperSNX24+4P reverse (5′pGATTCTGCTAGCTAGGC CTTAAACAAAA3′) formed SuperSNX24 linkers that were ligated to the digested DNA fragments. Linked DNA fragments were then amplified with SuperSNX24. Next, microsatellite markers including fragments with biotinated probes were isolated and enriched. The following three probe compositions were used: i) (TG)_12_, (ACT)_12_, (ACTG)_6_, and (ACAG)_6_; ii) (AG)_12_, (ACAT)_8_, (AACT)_8_, and (AAGT)_8_; and iii) (AAG)_8_, (AAAC)_6_, (AATC)_6_, and (AGAT)_6_. The biotinated probes were then annealed to fragments of gDNA containing complementary regions. Finally, the microsatellite-containing fragments were enriched using streptavidin-labeled metal beads (Dynabeads M-280 Streptavidin, catalog #11205D; Invitrogen, Waltham, MA, USA). The enriched segments were then cloned into pGEM-T Easy vector (pGEM-T Easy Vector system; Promega, Madison, WI, USA) and sequenced with an ABI 3730XL DNA Analyzer (Applied Biosystem PRISM, Waltham, MA, USA). The fragments with higher numbers of repeats were selected for polymorphism testing.

### Polymerase chain reaction and polymorphism testing

The selected highly repeated fragments were subjected to PCR and polymorphism testing, which would verify whether the microsatellite loci could be amplified and used to show diversity in the investigated populations. Primers for loci amplification were designed using Primer3plus. CAG-tag (5′-CAGTCGGGCGTCATCA-3′) or M13Reverse (5′-GGAA ACAGCTATGACCAT-3′) was added to the 5′ end of one of each primer pair. Following the protocol described by Schuelke [16], a fluorescent dye-labeled tag, as a third primer, was used with the primer pair to amplify the target fragments that were detectable upon capillary electrophoresis. Eight dog samples from four BIs and four SCs were tested at a 20-μL volume using a thermalcycler (GeneAmp PCR system 9700; Applied Biosystems, USA) containing 0.5 U Taq DNA polymerase (TAKARA, Kusatsu, Shiga, Japan), 1×PCR buffer (1.5 mM MgCl_2_), 0.2 mM deoxyribose nucleotide triphosphate (dNTP), 0.2 μM unlabeled primer, 0.04 μM tag-labeled primer, 0.16 μM dye-labeled tag, and 50 ng gDNA. The PCR cycling program was as follows: 95°C for 5 min, 35 cycles of 95°C for 30 s, 50°C to 65°C for 40 s, 72°C for 40 s, and a final elongation at 72°C for 7 min. The amplified microsatellite PCR products were analyzed with a DNA analyzer (ABI PRISM 3730 DNA analyzer; Applied Biosystems, USA). Allelic sizes of all loci were estimated relative to the in-line GeneScan500 LIZ Size Standard marker (ABI PRISM; Applied Biosystems, USA). The fragment size was calibrated and analyzed with Peak Scanner Software version 1.0 (ABI PRISM; Applied Biosystems, USA). Those loci that had an allele number greater than two and similar annealing temperatures were selected for whole-population analysis.

### Multiplex polymerase chain reaction

In total, 14 microsatellite loci were selected (Table 2). The 5′ end of each forward primer was labeled with the fluorescent dyes fluorescein (FAM), 2′-chloro-7′phenyl-1,4-dichloro-6-carboxy-fluorescein (VIC), red color fluorescent (PET), or 2′-chloro-5′-fluoro-7′,8′-benzo-1,4-dichloro-6-carboxyfluorescein (NED). Multiplex PCR was performed on a 30-μL reaction containing 1 U *Taq* DNA polymerase (TAKARA, Japan), 1×PCR buffer (1.5 mM MgCl_2_), 0.3 mM dNTP, 0.3 μM forward and reverse primer, and a 100-ng DNA template. The steps of the PCR program and genotype detection were as described above.

### Statistical analysis

For each locus and population, and across populations, commonly derived statistics from the microsatellite genotypic data, including allele frequencies, the observed number of alleles (No), the observed heterozygosity (Ho), the expected heterozygosity (H_E_), and the polymorphic information content (PIC), were calculated with the Microsatellite Toolkit. The Hardy–Weinberg equilibrium test was performed using the GENEPOP computer program [17], which also was used to estimate *F*-statistics (the global mean inbreeding coefficient [*F*_IT_], the average inbreeding coefficient of an individual with respect to the local subpopulation [*F*_IS_], and the average inbreeding coefficient of subpopulations relative to the total population [*F*_ST_]) for each locus, the pairwise *F*_ST_ between populations, and the average inbreeding coefficient (*F*_IS_). Nei’s genetic distance (D_A_) [18] between populations was measured with the Microsatellite Analyzer [19]. A phylogenetic tree was generated via the PHYLIP program [20] using the neighbor-joining (NJ) method with a bootstrap test of 1,000 resampling of loci with replacement [21].

The model-based approach proposed for the population structure analysis of the dog populations was carried out with the software STRUCTURE 2.3.1 [22], which was used to assess the genomic clustering (K) of the sample. To obtain a representative value of K for data modeling, 10 independent runs were performed for each value from one to seven. The run length was set to 100,000 burn-ins followed by 100,000 iterations. In addition, a principal coordinate analysis (PCoA) three-dimensional (3D) map drawn by GenAlEx [23] was used to confirm the inter-population situation.

The effectiveness of individual identification is expressed by the probability of identity (P_(ID)_), which is the probability that two individuals are randomly selected from the population and the genotypes of the two are identical at a single locus. The theoretical expectations are as follows:


$${\text{P}}_{\left(\text{ID}\right)}=\sum_{i=1}^{n}p_{i}^{4}+4\sum_{i=1}^{n}\sum_{j=1}^{n-1}p_{i}^{2}p_{j}^{2}$$

where *p_i_* and *p_j_* are the frequencies of the ith and jth alleles, and i ≠ j. When the molecular markers are co-dominant, such as microsatellite markers, and the identified individuals have close kinship, the following probability of identity among sibs (P_(ID)sib_) is applied:


$${\text{P}}_{(\text{ID})\text{sib}}=0.25+0.5\sum_{i=1}^{n}p_{j}^{2}+0.5\;{\left(\sum_{i=1}^{n}p_{j}^{2}\right)}^{2}-0.25\sum_{i=1}^{n}p_{j}^{4}$$

where *p_j_* is the frequency of the *j*th allele [24]. The calculation of the PE is also based on the formula mentioned by Jamieson [25].

## RESULTS

### Polymorphism, heterozygosity, and F-Statistics of novel microsatellite loci

The 14 microsatellite markers were used to perform genotyping on 113 samples from the three dog breeds (SCs, BIs, and BETs/BEJs). Polymorphism was clearly observed at most of the microsatellite loci in the three breeds. The genetic characteristics of the 14 microsatellite loci are listed in Table 3. The average number of alleles per locus (Na) was 6.3. The actual number of alleles ranged from 2 (SEL093 and SEL094) to 13 (SEL115). The average number of effective alleles per locus (Ne) ranged from 1.4 (SEL094) to 7.6 (SEL005), with an average across loci value of 3.6. The PIC value ranged from 0.249 (SEL094) to 0.855 (SEL005), with an overall average value of 0.612. All of the selected microsatellite loci in this study were sufficiently polymorphic, indicating that they were suitable for the genetic analysis of dogs.

The H_E_ among the 14 microsatellite loci ranged from 0.293 (SEL094) to 0.873 (SEL005), with an average of 0.662. The H_O_ among the 14 microsatellite loci ranged from 0.248 (SEL094) to 0.814 (SEL005), with an average of 0.567 (Table 3). However, there were eight loci—namely, SEL025, SEL030, SEL031, SEL035, SEL068, SEL098, SEL115, and SEL118—that significantly departed from the Hardy–Weinberg equilibrium (p<0.01).

The Wright’s *F*-statistic values (*F*_IS_, *F*_IT_, and *F*_ST_) for each locus are shown in Table 3. The average *F*_IS_ for all the loci was 0.002, and the *F*_IS_ per locus varied from −0.191 (SEL093) to 0.328 (SEL115). The average *F*_IT_ for all the loci was 0.209, and the *F*_IT_ per locus varied from −0.070 (SEL034) to 0.506 (SEL115). The mean *F*_ST_ for all the loci was 0.212. This value implied that around 21.2% of the total genetic variation was caused by population differences and that 78.8% of the total genetic variation was due to genetic differentiation among the individuals within each population.

### Intra–population genetic variability

The genetic statistics relating to polymorphism, including H_E_, H_O_, PIC, the mean observed number of alleles, and the mean effective number of alleles, were calculated to estimate the allelic diversity at each locus of the population. These genetic parameters across the 14 loci for the three dog populations are listed in Table 4. The H_E_ varied from 0.480 (SCs) to 0.624 (BEs), whereas the H_O_ varied from 0.485 (SCs) to 0.587 (BEs), and the PIC ranged from 0.407 (SCs) to 0.567 (BEs). The SC population had the lowest values of H_O_, H_E_, and PIC.

Among the three breeds, the BE population had the highest observed mean number of alleles (MNA) (5.3), followed by the BI (4.0) and SC (3.3) populations, while the latter had the smallest observed MNA. Negative *F*_IS_ values were observed in the BI and SC populations, indicating an insufficient degree of inbreeding. The deviation from the Hardy–Weinberg proportions within populations (*F*_IS_) varied from −0.032 to 0.045. The highest inbreeding effects were found in the BE population (0.045), which significantly deviated from the Hardy–Weinberg equilibrium (p<0.01) (Table 4) in comparison to the other two populations.

### Inter-population genetic variation

To estimate genetic variation among the five dog populations, two parameters—*F*_ST_ and genetic distance (D) calculated by the gene frequency of each animal at each microsatellite locus—were evaluated in this study. The values of *F*_ST_ and D for each test population pair are summarized in Table 5. The *F*_ST_ for each population pair was highly significant (p< 0.05). The *F*_ST_ values of the population pairs varied from 0.165 (for the BET and BEJB population pair) to 0.405 (for the SC and BEJB population pair). The genetic distances between the dog population pairs varied from 0.400 (for the BET and BEJB population pair) to 0.993 (for the SC and BEJA population pair). Surprisingly, the second relatively low genetic distance and *F*_ST_ value were not between other BE populations. The genetic distances and *F*_ST_ values of the BI–BET and SC–BET population pairs were less than the BEJA–BEJB and BEJA–BET population pairs.

### Population differentiation analysis

**Figure 1:**
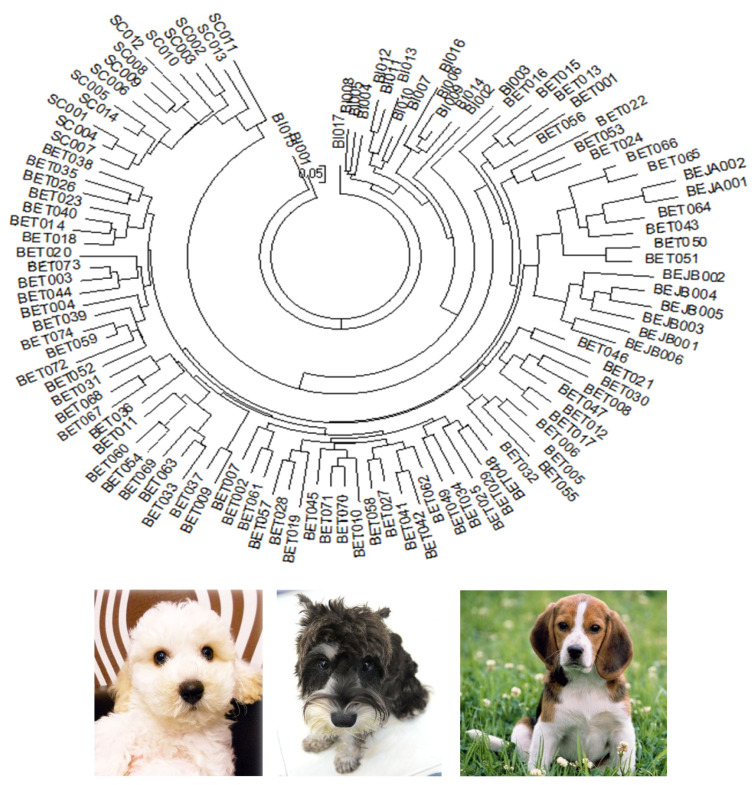
The neighbor-joining (NJ) tree among these dog individuals based on the 14 sets of novel microsatellite markers. SC, Schnauzer; BI, Bichon; BET, Beagle (Taiwan); BEJA, Beagle (Japan A); BEJB, Beagle (Japan B) in this study.

**Figure 2:**
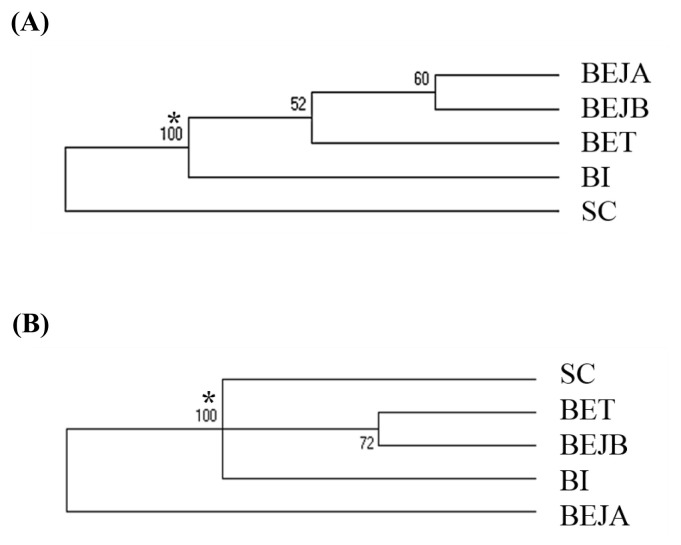
The (A) neighbor-joining (NJ) tree and (B) unweighted pair group method with arithmetic mean (UPGMA) tree among these dog populations based on the 14 sets of novel microsatellite markers. SC, Schnauzer; BI, Bichon; BET, Beagle (Taiwan); BEJA, Beagle (Japan A); BEJB, Beagle (Japan B). * Bootstrap values.

The Nei’s standard genetic distance of these three populations of dogs in Taiwan was calculated. A D_A_ distance matrix was used to build an individual phylogenetic tree with the NJ method (Figure 1). The results showed that the individual phylogenetic tree could be divided into three main clusters: SC, BI, and BE. Among these, the BE cluster could be further divided into a Taiwan population (BET) and two Japanese sub-populations: a Japan A (BEJA) and Japan B (BEJB) population. A phylogenetic tree using the NJ method with bootstrap resampling (n = 1,000) of the 14 microsatellite loci was constructed with the PHYLIP software. In the NJ tree (Figure 2A), the entire dog population could also be divided into three main clusters: SC, BI, and BE. The results of the main clusters were consistent with the results of the individual phylogenetic tree (Figure 1). In addition, the bootstrap value between the SC and other dog populations was 100%, which showed that the genetic distance between these groups was relatively large. Unlike the NJ method, the phylogenetic tree constructed by the unweighted arithmetic average pair group (UPGMA) method (Figure 2B) showed that the BEJA group was clearly distanced from others, with a bootstrap value of 100%. The remaining dog groups were divided into three clusters: SC, BI, and BET and BEJB.

**Figure 3:**
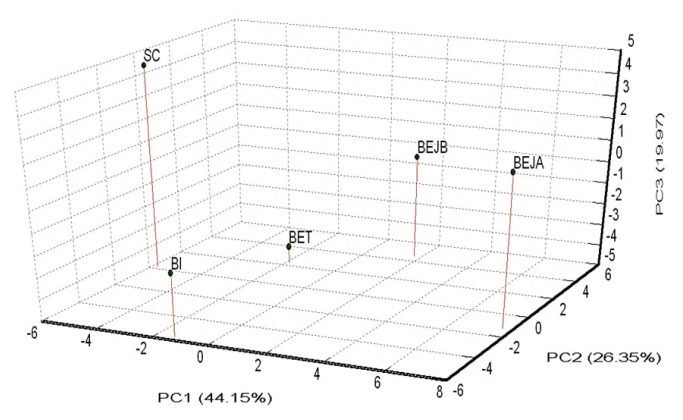
Principal coordinate analysis (PCoA) three-dimensional (3D) plot of dogs = relative to population genetic distances. SC, Schnauzer; BI, Bichon; BET, Beagle (Taiwan); BEJA, Beagle (Japan A); BEJB, Beagle (Japan B).

A PCoA of pair-wise genetic distances among the five examined dog sub-populations was used to represent the relative positions of the populations. The first (PC1), second (PC2), and third (PC3) PCos accounted for 44.15%, 26.35%, and 19.97% of the total variation, respectively (Figure 3). The distance between the BEJA and BEJB groups was relatively close. The distance between the BET group and both the BEJA and BEJB groups was closer than the distance between the BET group and both the SC and BI groups.

### Population structure analysis

**Figure 4:**
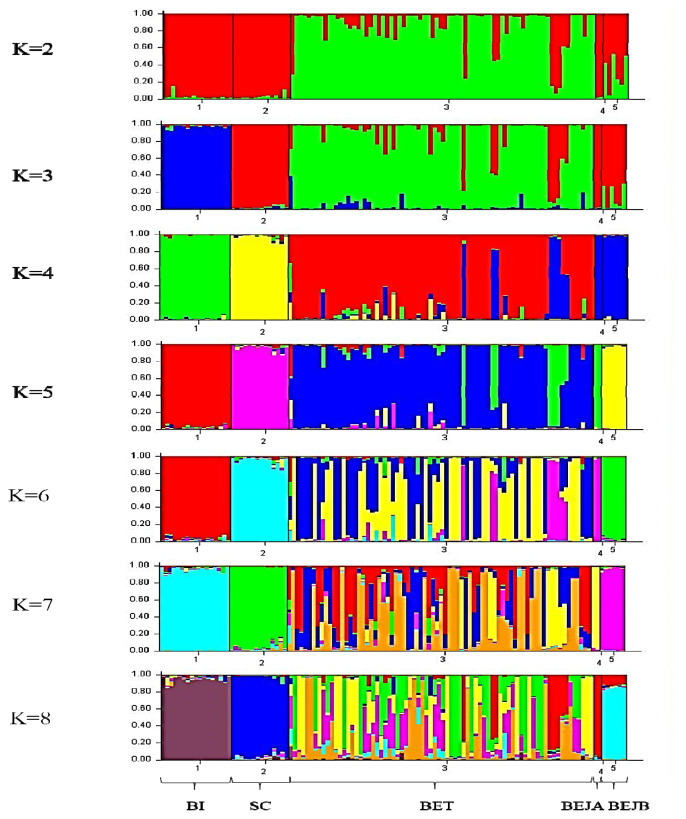
STRUCTURE cluster analysis plot (K = 2 to 8) of individual dogs based on the 14 sets of novel microsatellite markers. K is the number of clusters assumed in the STRUCTURE analysis, and the colors corresponded to clusters. The vertical axis indicates the proportion of gene resources in individuals from the clusters, and each individual is represented by a single bar. SC, Schnauzer; BI, Bichon; BET, Beagle (Taiwan); BEJA, Beagle (Japan A); BEJB, Beagle (Japan B).

The STRUCTURE software program using Bayesian model-based clustering algorithms of multi-locus genotypes was utilized to assign individuals to populations via estimated individual admixture proportions and to infer the number of populations (K) for a given sample. The results of the analysis are shown in Figure 4. It was mainly divided into Taiwan Beagle population and other populations at K = 2. When K = 3, the BI group was separated. At K = 4, it is mainly divided into BI, SC, BET, and BEJ four clusters. Until K = 5, the two BRJ clusters are separated finally. When the K value over 5, the BET populations are further subdivided into different clusters.

### Probability of identity and power of exclusion

The analysis of probability of identity involved three dog populations, respectively, and all populations together was calculated by the probability of identity (P_(ID)_) and the identification rate of close relatives (P_(ID)sib_) of the 14 single new microsatellite markers. The results are listed in Table 6 and Table 7. The P_(ID)_ of BE populations per locus varied from 0.042 (SEL005) to 0.491 (SEL094). The combined P_(ID)_ values for all loci was 3.7×10^−11^. The P_(ID)_ of each locus in BIs ranged from 0.060 (SEL005) to 1.000 (SEL094). The combined P_(ID)_ values for all loci was 7.8×10^−9^. The P_(ID)_ of SC populations per locus varied from 0.097 (SEL005) to 0.869 (SEL115). The combined P_(ID)_ values for all loci was 1.2×10^−7^. In addition, the P_(ID)_ of the 14 novel microsatellite loci for all dog populations ranged from 0.031 (SEL005) to 0.843 (SEL094), and the comprehensive P_(ID)_ was 1.7×10^−12^.

When individuals in the population to be explored have close relatives, such as full-sib or half-sib, it is more appropriate to evaluate the proportion of identification using the P_(ID)sib_. The P_(ID)sib_ of BE populations per locus varied from 0.337 (SEL005) to 0.701 (SEL094). The combined P_(ID)sib_ values for all loci was 3.9×10^−5^. In BIs, the P_(ID)sib_ ranged from 0.358 (SEL005) to 1.000 (SEL094), and the combined P_(ID)sib_ values for all loci was 2.6×10^−4^. The P_(ID)sib_ of SC populations per locus varied from 0.403 (SEL005) to 0.933 (SEL115). The combined P_(ID)sib_ values of SC for all loci was 7.1×10^−4^.

In the paternity test for dogs, according to the formula of power of exclusion (PE) proposed by Jamieson [25], the value calculated using the 14 new microsatellite markers’ allele frequencies was 99.98%. This meant that when the genotypes of the mother and offspring were known, the possibility of being a biological father could be almost completely eliminated for an individual who was not a sire.

## DISCUSSION

In this study, 14 sets of novel microsatellite markers were developed and used to analyze the genetic variation of three dog breeds—SC, BI, and BE. The results showed that the Na of the 14 novel microsatellite loci was 6.3, and the Ne was 3.6 (Table 3). The Na of locus SEL115 was 13, whereas the Ne was only 3.8. The reason might have been that the distribution of allele frequency was mainly concentrated on three alleles—243 bp (13.3%), 247 bp (10.6%), and 251 bp (47.4%) (Supplementary Table S1)—such that in some cases the uneven distribution of allele frequencies caused a large gap between the Na and the Ne.

The H_E_, H_O_, and PIC are commonly used to assess the polymorphism of microsatellite loci in the analyzed population. The H_O_ refers to the observed heterozygosity of each locus, which represents the actual proportion of heterozygous individuals in the population. The H_E_ is the expected heterozygosity of each locus, which is the expected proportion of heterozygous individuals in the population that is calculated according to the Hardy–Weinberg Law. The PIC is the degree of polymorphism of each locus. Using the 14 novel microsatellite markers to analyze our dog populations, the average H_E_ was 0.662, which showed that the values of most of the microsatellite markers were within high expected heterozygosity (H_E_>0.5). The average value of H_O_ was 0.567, which also fell within the range of high observed heterogeneity (0.7>H_O_>0.5) [26]. The average value of PIC was 0.612, which fell within the range of high polymorphic information content (PIC>0.5) [27] (Table 3). The results of this experiment were similar to the study of Radko et al [28] using 18 sets of microsatellite markers to analyze the Polish Tatra shepherd dog. Their results showed that the average H_E_ was 0.643, the average H_O_ was 0.645, the average PIC was 0.598, and the values of the three variables were all greater than 0.5. Therefore, their study indicated that the tested Tatra shepherd dog population was highly polymorphic. In another study [29], eight breeds of dog were surveyed by 21 microsatellite markers. PIC values over 0.5 were measured for 15 markers. The average value of the PIC was 0.555. Compared with these reports, the three variables in the current experiment were highly polymorphic, indicating that the 14 novel microsatellite loci should be able to effectively analyze the genetic structure and genetic variation of the three breeds of dogs analyzed in this experiment.

The H_E_ (0.412 and 0.293) and H_O_ (0.451 and 0.248) values of loci SEL093 and SEL094 were both less than 0.5, as were the respective PIC (0.326 and 0.249) values. The cause of this result, for which there were only two alleles in two loci, was supposed to be sampling error. However, some reports have suggested that the number of alleles for microsatellite markers should be three or more to reduce the standard deviation of distance calculation [30]. The reason why the loci SEL093 and SEL094 were selected in this experiment was that the number of dog breeds analyzed was relatively small. If the number of breeds is increased, perhaps the allele number of these two microsatellite loci could be increased, and the three variables will be likely to increase as well. On this basis, the two microsatellite loci SEL093 and SEL094 were retained as potential canine microsatellite loci in this experiment.

In terms of the analysis of population genetic structure, we applied the *F*_IS_, *F*_ST_, and *F*_IT_ statistics to evaluate the distribution of genetic variation within and between populations. The average *F*_IS_ value of the 14 new microsatellite markers was 0.002. This value was positive and low. The percentage of heterozygotes in the overall tested dog population was less than expected—that is, there was an inbreeding phenomenon—but the average value of *F*_IS_ was around 0.002, which indicated that the situation in the dog population was not serious. The mean *F*_ST_ for all the loci was 0.212. This fell within the range of high differentiation (0.15<*F*_ST_<0.25), according to the Sewall Wright rules [31], indicating that there was high differentiation among the three breeds of dogs in this study.

Kang et al [32] investigated the genetic structure of local dogs in South Korea and establish an individual and paternity identification system through evaluating the polymorphisms of the populations with three variables: H_O_, H_E_, and PIC. Between nine and 11 microsatellite loci were used for genetic analysis of two local breeds from South Korea and three exotic dog breeds in their study. The sample selection criterion was at least one generation of unrelated dog individuals. The results showed that the average H_O_ for each breed ranged from 0.65 to 0.78; the H_E_ ranged from 0.71 to 0.85; and the PIC ranged from 0.66 to 0.82. In this study, the average value of H_E_ for the three varieties ranged from 0.480 (SCs) to 0.624 (BEs), and average H_O_ ranged from 0.485 (SCs) to 0.587 (BEs). The average PICs ranged between 0.407 (SCs) and 0.567 (BEs) (Table 4). Compared with the abovementioned studies, the three variables in our experiment showed slightly lower values, which may have been caused by differences in the individual dogs included in this experiment: some of the animals were blood-relatives and were full-sibs or half-sibs, so their genetic backgrounds were similar, leading to slightly lower polymorphisms. Future testing of individual Taiwanese dogs with different origins or more distant blood relationships could improve the applicability of these new microsatellite markers.

Among the different varieties of *F*_IS_, only the average value for BEs (0.045) was positive. The results showed that although BEs in this experiment had high genetic variation, the positive value of *F*_IS_ indicated that the proportion of heterozygous individuals was still too small to achieve the Hardy–Weinberg balance; Iindeed, it deviated significantly from the expected value (p<0.01). This result may reflect the fact that the fathers of the BE population in this experiment comprised a small number of male dogs, which was not reflective of the situation of mating by chance. The BIs and SCs did not deviate from the Hardy–Weinberg equilibrium, and the *F*_IS_ values of these two breeds were negative. It can thus be inferred that these two breeds had no inbreeding issues, the genetic backgrounds of the parents were different, and the number of male and female animals was equal [33]. Therefore, it was supposed that the deviation of the entire dog population from the Hardy–Weinberg balance was attributable to the deviation of the BE population.

The individual phylogenetic tree of the dogs (Figure 1) was constructed by the NJ method, and the cluster analysis diagram drawn by the STRUCTURE software (Figure 4), and the dogs in this experiment were divided into five groups: SC, BI, BET, BEJA, and BEJB. In the phylogenetic tree of dog populations drawn by the NJ method (Figure 2A), the distinctive main clusters were consistent with the result of the individual phylogenetic tree: both could clearly distinguish the BI, SC, and BE groups, and the BET and BEJ groups were closely identified. The different breeds of dogs could be clearly differentiated by the microsatellite markers used in this experiment. The differentiation of dog breed in tree would be caused by the unique alleles. For example, the alleles 185 bp and 201 bp of the SEL035 locus were only found in the BI and SC populations, respectively (Supplementary Table S1). In the phylogenetic tree drawn by the NJ method, the bootstrap value of the BET and the BEJ group was 52%, which means that only 52% of the analysis results separated them. When the bootstrap value between the two populations was not greater than 70% in neighbor joining tree, it showed that the clustering of that two populations were not obvious [34]. In the NJ phylogenetic tree, the bootstrap value between the SC and other dog populations was 100%, and the result was in the highly reliable range (bootstrap value>70%). That showed that the genetic distance between the SC and other dog populations was relatively long. However, the UPGMA phylogenetic tree (Figure 2B) shows that the BEJA group was far away from other dog groups, and the bootstrap value was 100%. But the UPGMA method based on constant-rate assumption, and the distance between samples on the same branch is the same, so this method is only suitable for the case where all samples have the same evolution distance [35], so recently it was less used to construct a phylogenetic tree, and the NJ method was a more commonly used method for drawing a phylogenetic tree.

The PCoA draws a 3D stereogram based on the genetic distance between the populations (Figure 3), and distinguishes the genetic distance among the populations by the variance of the three principal coordinate axes. It can be found that the two BEJ groups were relatively close, which was consistent with the close geographical relationship between the two, and the relative distance between the two groups and the BET group was closer than the relative distance between the two groups and the SC and the BI. The results of the NJ phylogenetic tree can also be confirmed in the 3D map of the PCoA. The SC and the BEJA were located on the farthest sides of the 3D map, so there was a maximum genetic distance between the two populations.

In the individual analysis, the combined probability of identification (P_(ID)_) of the 14 new microsatellite loci for the entire dog population was 1.7×10^−12^ (Table 6), and according to the survey of the Council of Agriculture up until the end of 2019, the total number of dogs in Taiwan was about 1.54×10^6^ [36]. That meant when the current number of Taiwanese dog populations is analyzed by the 14 new microsatellite markers used in this experiment, the probability of appearing exactly the same genotype is very low. Although the dog population in this experiment contained only three breeds, however, when examining P_(ID)_ of a single dog breed, the credibility is still high. There are no statistics on the numbers of dogs of different breeds currently in Taiwan; however, it can be ascertained that the number of dogs in any single breed cannot exceed the total number of dogs. Therefore, the P_(ID)_ of the three dog populations respectively in this study should cover the total number of dogs in Taiwan in 2019. It was confirmed that the probability of the same genotype being identified in two individuals with 14 the sets of novel microsatellite markers was very low.

The combined probability of identity among sibs (P_(ID)sib_) was 1.6×10^−5^ (Table 7) in this study. According to Waits et al [24], their markers were sufficient to identify close relatives of the natural population when the P_(ID)sib_ was between 10^−3^ and 10^−4^. The P_(ID)sib_ values of the three breeds of dogs in this experiment generally met this recommended standard. This confirmed that the 14 novel microsatellite markers were applicable in the individual identification of close relatives of BEs, BIs, and SCs in Taiwan.

In the paternity tests of dogs, the PE of the 14 new microsatellite markers in this experiment was 99.98%, which meant that when the genotypes of the mother and offspring were known, individuals that were not the biological father could be ruled out. According to the pedigree data for the BE population collected in this experiment, there was one of the cases where the mother–child genotypes were known, the individual registered as the biological father in the pedigree was compared with the genotypes of 14 novel microsatellite markers and which was not found to provid any of the alleles to the offspring at six of the loci. Therefore, it could be inferred that the male was not the biological father, and suggested that the registration of the dog’s pedigree data was inaccurate. This supports the suggestion that many dog-breeding facilities in Taiwan are still not rigorous enough for pedigree registration. Therefore, using paternity facilities with high PE to improve the registration of pedigree in Taiwan is important. The microsatellite markers developed in this study may be suitable for this purpose to avoid the potential damage caused by inbreeding.

At present study, many countries used microsatellite markers to analyze dog populations. For developing a platform of paternity and individual identification, the American Kennel Club (AKC) analyzed 108 dog breeds using 17 microsatellite markers. The results show that the average H_O_ was 0.60, and the average value of PIC was 0.56. PE was more than 99% in all breeds, and the combined P_(ID)_ was 3.2×10^−8^. The American Kennel Association considered that 17 microsatellite markers were sufficient for ordinarily paternity identification [37]. Eichmann et al. established Austrian dog DNA profiling for investigation of dog-related accidents and crimes, using 15 sets of highly polymorphic microsatellite markers to analyze 45 dog breeds [38]. The results revealed that the average H_O_ was 0.74, the average PIC was 0.82, and the combined P_(ID)sib_ was 8.5×10^−8^, showing that these 15 microsatellite markers are sufficient for individual identification of dogs in Austria. Kang et al [32] used 9 to 11 sets of microsatellite markers to detect the genetic structure of local dogs in South Korea and established an individual and paternity identification system to perform genetic analysis of two local breeds of South Korea and three foreign dog breeds. The results showed that the average H_O_ for each breed was between 0.65 and 0.78; the average PIC was between 0.66 and 0.82; and the average PE was more than 99% in all breeds. The average H_O_ value (0.57) of the 14 new microsatellite markers in this study near the range of the research results of the previous countries (0.60 to 0.74). The average value of PIC (0.61) was also within the range of the results of the aforementioned countries (0.56 to 0.82). According to the researches before, the 14 new microsatellite markers developed in this study were highly polymorphic and suitable to analyze the three breeds of dogs in Taiwan.

In conclusion, using 14 novel microsatellite markers to analyze the beagle, bichon, and schnauzer populations in Taiwan, the results showed that their average expected heterozygosity, observation heterozygosity, and polymorphism information content were all at high levels. Therefore, these new microsatellite markers have high applicability to the analyzed populations. These results indicate that the new microsatellite markers have good resolution when applied to the detection of differences among dog breeds. It was confirmed that the opportunity of identifying the exact same genotype among the analysis of the 14 new microsatellite markers was very low. In addition, the power exclusion was enough high to be a good tool for paternity testing.

## Figures and Tables

**Table 1:** Number of dogs of the three breeds from different farms

Breed	Abbreviation^1)^	Farm^2)^	Sample size
Bichon	BI	K	17
Schnauzer	SC	K	14
Beagle	BET	P	74
	BEJA	P	2
	BEJB	NTU	6
Total			113

1) BET, beagle bred in Taiwan; BEJA and BEJB, beagle imported from Japan.

2) K farm located in Kaohsiung city in Taiwan; P farm located in Pingtung county in Taiwan; NTU, National Taiwan University.

**Table 2:** Primer sequences, repeated motifs, and fluorescent labeling of 14 microsatellite loci

Locus	Primer sequence (5′–3′)	Repeat motif	Size (bp)	Label
SEL005	F: AGCTTGCCAACTTCACTCGT R: TCCTGCTTGGTCTCCTGATT	(CTTTT)_17_	186–260	FAM
SEL105	F: TCAACAATTGAAATATACAAGTTAGCA R: GGGCATGATCCTAGAGTCCA	(GA)_10_	166–172	FAM
SEL117	F: CAGCCTGGAGATACACAGCA R: CCACTGGAAACACAGCAGAA	(TG)_17_	147–153	FAM
SEL030	F: TGGAGACTCGGGATCAAATC R: CCTACCCATTTCGCTCATGT	(TC)_18_	189–209	NED
SEL031	F: GACCATCTCCATTGAGAACCA R: TACAACGGTCTTTCCCAGGT	(CT)_13_	159–175	NED
SEL068	F: CTTGCCCCTGAGCAAGATAC R: GGTGTGTCCGCCTTAAAGAA	(CA)_12_	160–201	VIC
SEL098	F: ATACAGTTGGTGCCCAAAAA R: CTCCCTGCTCACACACACAC	(GA)_8_(TG)_10_	226–236	VIC
SEL034	F: GCTTCTCACATGCAACATGG R: GGCCTCCCAAGAAATGGTAT	(CA)_11_	187–193	PET
SEL094	F: GACCATCTCCAGCCATCCTA R: TGGGTTTGAATTGGCTAACA	(TC)_14_	187–191	FAM
SEL115	F: TCACAAATGGCAAAATCTTTCTT R: AAGCAACCCAAGTGTCCATC	(GATA)_19_(GGTA)_18_	203–263	FAM
SEL035	F: CACTGAGCATCCACTGAAGG R: CACCCACCATGGCTCTCTAT	(TG)_15_(AG)_11_	185–211	NED
SEL118	F: CTGGGCTGGGTAGTCTGTTC R: TCCCCCAAGTGATTCTTCTG	(CA)_19_	159–172	NED
SEL025	F: GCACAGGCTTTTCATATCCA R: AATGAGTGAATGGGCACCTC	(CT)_14_(CA)_14_	143–157	PET
SEL093	F: GTGGTAGGGAGAGGGACAGA R: CTCCTGCTGACCTTTCTTGG	(GA)_19_	174–176	PET

FAM, blue color fluorescent; NED, yellow color fluorescent; VIC, green color fluorescent; PET, red color fluorescent.

**Table 3:** Genetic variability of the total dog population from three dog breeds genotyped with 14 sets of novel microsatellite markers

Locus	*F*_IS_^1)^	*F*_ST_^1)^	*F*_IT_^1)^	N_a_^1)^	N_e_^1)^	H_O_^1)^	H_E_^1)^	PIC^1)^	Exact test of HWE^2)^
SEL005	0.001	0.119	0.119	10	7.6	0.814	0.873	0.855	NS
SEL025	−0.013	0.244	0.234	8	4.4	0.670	0.775	0.741	^*^
SEL030	0.092	0.249	0.318	9	4.5	0.602	0.779	0.741	^*^
SEL031	0.135	0.340	0.429	5	3.5	0.487	0.717	0.667	^*^
SEL034	−0.091	0.019	−0.070	3	2.2	0.602	0.557	0.489	NS
SEL035	0.002	0.295	0.297	10	5.2	0.664	0.813	0.791	^*^
SEL068	−0.104	0.161	0.073	7	2.9	0.655	0.654	0.608	^*^
SEL093	−0.191	0.138	−0.027	2	1.7	0.451	0.412	0.326	NS
SEL094	−0.010	0.266	0.258	2	1.4	0.248	0.293	0.249	NS
SEL098	−0.116	0.355	0.281	6	3.8	0.637	0.739	0.695	^*^
SEL105	−0.041	0.078	0.040	4	2.4	0.584	0.587	0.494	NS
SEL115	0.328	0.265	0.506	13	3.8	0.416	0.737	0.714	^*^
SEL117	−0.034	0.132	0.103	3	2.3	0.549	0.574	0.481	NS
SEL118	0.077	0.306	0.359	6	4.1	0.566	0.757	0.714	^*^
Mean	0.002	0.212	0.209	6.3	3.6	0.567	0.662	0.612	

1) *F*_IS_, Wright’s F-statistics, within subpopulation inbreeding estimate; *F*_ST_, Wright’s F-statistics, among subpopulation differentiation estimate; *F*_IT_, Wright’s F-statistics, within total population inbreeding estimate; N_a_, number of alleles; N_e_, effective number of alleles; H_o_, observed heterozygosity; H_E_, expected heterozygosity; PIC, polymorphism information content.

2) Hardy-Weinberg equilibrium.

* Significant (p<0.01) departure from the Hardy–Weinberg equilibrium.

NS, not significant.

**Table 4:** Genetic parameters across 14 loci in the three dog populations

Breed	Sample size	MNA		Mean heterozygosity		PIC	FIS	HWE test
	
		Observed	Effective	Observed (H_O_)	Expected (H_E_)			
Beagle	82	5.3±2.7	3.0±1.2	0.587±0.122	0.624±0.133	0.567±0.149	−0.045±0.174	^*^
Bichon	17	4.0±2.0	2.5±1.1	0.542±0.271	0.531±0.232	0.497±0.212	−0.032±0.246	NS
Schnauzer	14	3.3±1.6	2.1±0.9	0.485±0.251	0.480±0.206	0.407±0.191	−0.001±0.210	NS
Total	113	6.3±3.4	3.6±1.6	0.567±0.136	0.662±0.162	0.612±0.179	−0.002±0.128	

MNA, mean number of alleles; PIC, polymorphism information content; *F*_IS_, the measure of the deviation from the Hardy–Weinberg proportions within subpopulations; HWE, Hardy-Weinberg equilibrium;

* p<0.01;

NS, not significant.

**Table 5:** Pair-wise estimates of breed differentiation (*F*_ST_) (above the diagonal) and genetic distance (D) (below the diagonal) between each pair of the five dog populations

Population	BI	SC	BET	BEJA	BEJB
BI	-	0.274	0.207	0.306	0.281
SC	0.523	-	0.227	0.405	0.287
BET	0.461	0.491	-	0.274	0.165
BEJA	0.701	0.993	0.788	-	0.262
BEJB	0.689	0.608	0.400	0.585	-

*F*_ST_: Wright’s F-statistics, among population differentiation estimates.

BI, bichon; SC, schnauzer; BET, beagle (Taiwan); BEJA, beagle (Japan A); BEJB, beagle (Japan B).

**Table 6:** The probability of identity (P_(ID)_) of 14 sets of novel microsatellite markers in different dog breeds and the total dog population

Locus	Beagle	Bichon	Schnauzer	Total population
SEL005	0.042	0.060	0.097	0.031
SEL025	0.149	0.161	0.250	0.083
SEL030	0.143	0.264	0.342	0.085
SEL031	0.164	0.277	0.578	0.128
SEL034	0.240	0.323	0.461	0.264
SEL035	0.116	0.188	0.113	0.054
SEL068	0.222	0.163	0.211	0.164
SEL093	0.441	0.717	0.387	0.432
SEL094	0.491	1.000	0.600	0.843
SEL098	0.170	0.593	0.149	0.110
SEL105	0.286	0.191	0.343	0.263
SEL115	0.094	0.158	0.869	0.091
SEL117	0.298	0.248	0.497	0.275
SEL118	0.137	0.353	0.434	0.101
Combined	3.7×10^−11^	7.8×10^−9^	1.2×10^−7^	1.7×10^−12^

P_(ID)_, probability of identity.

**Table 7:** The probability of identity among sibs (P_(ID)sib_) of 14 sets of novel microsatellite markers in different dog breeds and the total dog population

Locus	Beagle	Bichon	Schnauzer	Total population
SEL005	0.337	0.358	0.403	0.323
SEL025	0.444	0.451	0.533	0.385
SEL030	0.432	0.525	0.600	0.383
SEL031	0.468	0.536	0.768	0.425
SEL034	0.519	0.576	0.678	0.538
SEL035	0.428	0.481	0.412	0.359
SEL068	0.509	0.466	0.490	0.466
SEL093	0.661	0.849	0.608	0.653
SEL094	0.701	1.000	0.778	0.740
SEL098	0.472	0.773	0.445	0.410
SEL105	0.539	0.468	0.584	0.524
SEL115	0.405	0.469	0.933	0.406
SEL117	0.547	0.522	0.706	0.533
SEL118	0.439	0.592	0.654	0.398
Combined	3.9×10^−5^	2.6×10^−4^	7.1×10^−4^	1.6×10^−5^

P_(ID)sib_, probability of identity among sibs.
