# Supplementary material for: Development of novel microsatellite markers to analyze the genetic structure of dog populations in Taiwan
Source: Anim Biosci. 2022 Mar 2;35(9):1314–26. doi: 10.5713/ab.21.0519 (PMC9449399; doi:10.5713/ab.21.0519)
Supplement: Supplementary file 1 [file ab-21-0519-suppl.pdf]

Supplementary material – Allele frequency of each marker in dog population

Table S

Alleles and allelic frequency (%) of different dog breeds and the total dog population by 14 sets of novel microsatellite markers

		Beagle	Bichon	Schnauzer	Total
SEL005	186	20.1	2.9	7.1	15.9
	204	4.3	-	7.1	4.0
	209	-*	20.6	-	3.1
	232	14.6	23.5	7.1	15.0
	235	4.9	5.9	42.9	9.7
	240	5.5	5.9	-	4.9
	245	20.7	8.8	-	16.4
	250	11.6	5.9	21.4	12.0
	255	1.8	-	10.7	2.7
	260	16.5	26.5	3.6	16.4
SEL025	143	-	2.9	-	0.5
	145	29.6	-	-	21.4
	147	5.6	-	60.7	11.6
	149	44.4	23.5	7.1	36.6
	151	-	2.9	-	0.5
	153	15.4	26.5	-	15.2
	155	3.1	44.1	7.1	9.8
	157	1.9	-	25.0	4.5
SEL030	189	1.2	-	-	0.9
	193	1.2	47.1	-	8.0
	195	28.7	-	25.0	23.9
	197	33.5	2.9	-	24.8
	199	-	2.9	67.9	8.9
	201	31.1	44.1	7.1	30.1
	203	0.6	-	-	0.4
	205	3.7	-	-	2.7
	209	-	2.9	-	0.4

Table S

Alleles and allelic frequency (%) of different dog breeds and the total dog population by 14 sets of novel microsatellite markers (continued)

		Beagle	Bichon	Schnauzer	Total
SEL031	159	10.4	-	-	7.5
	167	9.8	8.8	-	8.4
	171	18.9	38.2	85.7	30.1
	173	54.3	-	10.7	40.7
	175	6.7	52.9	3.6	13.3
SEL034	187	18.3	5.9	-	14.2
	189	25.0	32.4	25.0	26.1
	193	56.7	61.8	75.0	59.7
SEL035	185	-	11.8	-	1.8
	189	9.1	2.9	7.1	8.0
	191	15.9	5.9	7.1	13.3
	193	0.6	26.5	3.6	4.9
	195	6.1	-	14.3	6.2
	197	6.1	-	-	4.4
	201	-	-	28.6	3.5
	203	7.9	52.9	-	13.7
	205	50.0	-	-	36.3
	211	4.3	-	39.3	8.0
SEL068	160	57.3	52.9	25.0	52.7
	162	3.0	2.9	50.0	8.9
	189	-	14.7	-	2.2
	191	19.5	-	-	14.2
	193	20.1	20.6	25.0	20.8
	195	-	2.9	-	0.4
	201	-	5.9	-	0.9

Table S

Alleles and allelic frequency (%) of different dog breeds and the total dog population by 14 sets of novel microsatellite markers (continued)

		Beagle	Bichon	Schnauzer	Total
SEL093	174	27.4	8.8	60.7	28.8
	176	72.6	91.2	39.3	71.2
SEL094	187	22.0	-	14.3	17.7
	191	78.0	100.0	85.7	82.3
SEL098	226	11.0	-	-	8.0
	228	4.3	14.7	25.0	8.4
	230	54.3	-	3.6	39.8
	232	10.4	85.3	17.9	22.6
	234	20.1	-	46.4	20.4
	236	-	-	7.1	0.9
SEL105	166	45.1	35.3	60.7	45.6
	168	47.6	38.2	35.7	44.7
	170	7.3	26.5	-	9.3
	172	-	-	3.6	0.4
SEL115	203	4.3	-	-	3.1
	207	3.7	2.9	-	3.1
	211	0.6		-	0.4
	215	2.4	-	-	1.8
	227	6.1	-	-	4.4
	235	-	2.9	3.6	0.9
	239	9.1	2.9	-	7.1
	243	18.3	-	-	13.3
	247	3.0	55.9	-	10.6
	251	45.7	14.7	96.4	47.4
	255	0.6	-	-	0.4
	259	6.1	8.8	-	5.8
	263	-	11.8	-	1.8

Table S

Alleles and allelic frequency (%) of different dog breeds and the total dog population by 14 sets of novel microsatellite markers (continued)

		Beagle	Bichon	Schnauzer	Total
SEL117	147	48.8	14.7	21.4	40.3
	151	6.1	29.4	-	8.9
	153	45.1	55.9	78.6	50.9
SEL118	159	0.6	-	-	0.4
	164	47.6	2.9	-	35.0
	166	9.1	61.8	71.4	24.8
	168	11.6	35.3	28.6	17.3
	170	7.3	-	-	5.3
	172	23.8	-	-	17.3

-: Not detected.
